# Clinical, hematological, biochemical, and histopathological evaluations in domestic cats (*Felis catus*) infected by *Leishmania infantum*

**DOI:** 10.1590/S1984-29612023037

**Published:** 2023-07-07

**Authors:** Joilson Ferreira Batista, Francisco das Chagas Ribeiro Magalhães, Kayo Sandro Pimentel do Prado Lopes, Carla Menezes Guimarães Sousa, Diana Sousa Alcântara, Sílvia de Araújo França Baêta, Michel Muálem de Moraes Alves, Ivete Lopes de Mendonça

**Affiliations:** 1 Programa de Pós-graduação em Ciência Animal, Universidade Federal do Piauí - UFPI, Teresina, PI, Brasil; 2 Laboratório de Sanidade Animal, Universidade Federal do Piauí - UFPI, Teresina, PI, Brasil; 3 Departamento de Clínica e Cirurgia Veterinária, Universidade Federal do Piauí - UFPI, Teresina, PI, Brasil; 4 Departamento de Morfofisiologia Veterinária, Universidade Federal do Piauí - UFPI, Teresina, PI, Brasil

**Keywords:** Leishmania infantum, feline, blood count, leukogram, histopathology, Leishmania infantum, felino, hemograma, leucograma, histopatologia

## Abstract

A high frequency of feline leishmaniasis has been reported in several countries. However, much information about disease progression in cats still needs to be clarified. This study aimed to verify the occurrence of clinicopathological changes in cats infected with *Leishmania infantum.* A total of 60 cats were divided into three groups of 20 animals each: control, suspects, and infected. All 60 cats underwent blood count and biochemical analyses. Serum samples from 20 animals with leishmaniasis were also used to diagnose feline immunodeficiency virus and feline leukemia virus. A total of five of the infected animals underwent necropsy for a histopathological study. The main clinical findings in cats with leishmaniasis were lymphadenomegaly (65%), alopecia (55%), ulcerative skin lesions and weight loss (40%), skin nodules (25%), a significant reduction in red blood cells (p=0.0005) and hematocrit (p=0.0007), hyperplasia in spleen 4/5(80%), presence of *Leishmania* in the spleen 2/5(40%), hepatitis 3/5(60%), liver degeneration 4/5(80%) and inflammatory nephropathy 3/5(60%). It was concluded that cats with leishmaniasis presented significant clinical, hematological, and histopathological alterations compatible with *L. infantum* infection. The observation of lymphadenomegaly, weight loss, skin lesions and low concentration of red blood cells, contributes significantly to the diagnosis and analysis of progression of feline leishmaniasis.

## Resumo

Uma alta frequência de leishmaniose felina tem sido relatada em vários países. No entanto, muitas informações sobre a progressão da doença em gatos ainda precisam ser esclarecidas. Este estudo teve como objetivo verificar a ocorrência de alterações clinicopatológicas em gatos infectados por *Leishmania infantum*. Um total de 60 gatos foi dividido em três grupos de 20 animais cada: controle, suspeitos e infectados. Todos os 60 gatos foram submetidos à hemograma e testes bioquímicos. Amostras de soro de 20 animais com leishmaniose também foram utilizadas para diagnosticar o vírus da imunodeficiência felina e o vírus da leucemia felina. Um total de cinco dos animais infectados foram necropsiados para estudo histopatológico. Os principais achados clínicos nos gatos com leishmaniose foram linfadenomegalia (65%), alopecia (55%), lesões ulcerativas na pele e emagrecimento (40%), nódulos cutâneos (25%), redução significativa de hemácias (p=0,0005) e hematócrito (p=0,0007), hiperplasia no baço 4/5(80%), presença de *Leishmania* no baço 2/5(40%), hepatite 3/5(60%), degeneração hepática 4/5(80%) e nefropatia inflamatória 3/5(60%). Concluiu-se que gatos com leishmaniose apresentaram alterações clínicas, hematológicas e histopatológicas significativas compatíveis com infecção por *L. infantum*. A observação de linfadenomegalia, emagrecimento, lesões cutâneas e baixa concentração de hemácias, contribui significativamente para o diagnóstico e análise da progressão da leishmaniose felina.

## Introduction

In Brazil, visceral leishmaniasis (VL) is caused by *Leishmania infantum,* which is responsible for causing VL in Americans with the dog as the main reservoir (Harhay et al., 2011; Marcondes & Rossi, 2013). Infection by *L. infantum* affects both humans and several domestic and wild animal species (Harhay et al., 2011; Zanette et al., 2014). It has been reported in cats from several countries, occurring mainly in endemic areas for canine and human VL. Since VL is endemic in Teresina, Piauí, Brazil, *L. infantum* infection has been reported in cats (Mendonça et al., 2017).

VL is known to be a serious, systemic infectious disease that can be fatal if left untreated (Silva, 2007). However, infection by *L. infantum* in cats is still poorly studied, leading to a lack of information regarding the pathogenesis of leishmaniasis in cats. These animals develop an efficient immune response against *L. infantum,* presenting a natural resistance, that, due to genetic factors, inhibits the development of parasites (Costa et al., 2010). Several studies have reported infection in cats, with many of them presenting severe clinical signs. However, clinical alterations are manifested mainly by immunodeficient animals (Pennisi, 2015; Pennisi et al., 2015).

In a study by Navarro et al. (2010), skin changes (ulcers and nodules), visceral involvement (hepatic, splenic, and renal alterations), and ocular signs (conjunctivitis, blepharitis, and/or keratitis) were observed. However, hematological and biochemical quantifications in cats with leishmaniasis are still poorly understood, and no published study has analyzed a possible difference between groups of infected and healthy animals using these clinical parameters.

Due to the increasing number of reports of leishmaniasis in cats and the lack of information about the clinical profile of the disease in these animals (Pennisi et al., 2015), the objective of this study was to evaluate the clinical signs and hematological, biochemical, and histopathological changes, which may eventually be present in domestic cats with leishmaniasis and also to assess the interference of *Retrovirus* infection in clinical manifestations of cats with leishmaniasis.

## Material and Methods

### Location

The study was conducted at the Animal Health Laboratory (LASAN) and the Animal Pathology Laboratory, both located at the Agrarian Sciences Center (CCA) at the Federal University of Piauí (UFPI).

### Experimental design

A total of 60 domestic cats (*Felis catus*), all domiciled, from a parallel study carried out in Teresina, Piauí, Brazil, which detected 20 out of 307 cats to be infected by *L. infantum,* were included in this study. Polymerase chain reaction (PCR) was used to confirm *L. infantum* infection, using specific primers to amplify 300 to 350 bp fragments of the *L. infantum ITS1* gene, followed by restriction fragment length polymorphism (RFLP) analysis, using the HaeIII enzyme (Mendonça et al., 2017).

For this study, 20 animals positive for *Leishmania infantum* were used, diagnosed by direct parasite search in a parasitological examination of bone marrow, popliteal lymph node, or skin (infected group); 20 animals with clinical alteration or positive serology, but negative by direct search of *Leishmania* sp*.* were randomly selected (suspected group), and 20 animals negative by serological and parasitological examination for leishmaniasis and without any alteration during clinical evaluation (control group).

All 60 cats were subjected to blood count and biochemical measurements. Blood serum samples from the 20 animals with positive parasitological examination were subjected to *Retrovirus* testing (feline immunodeficiency virus and feline leukemia virus). Of the total samples, five were donated by the owners for the study and underwent necropsy to check for possible changes in organs. Spleen and liver aspiration was performed on them to search for the parasite, and fragments of the spleen, liver, and kidney were collected for histopathological evaluation.

### Parasitological and serological examination for the diagnosis of leishmaniasis

Popliteal lymph node, bone marrow, spleen, and liver samples underwent direct search for *Leishmania* sp*.* using culture medium seeded in tubes containing Novy, MacNeal, Nicolle (NNN) biphasic culture medium and 1 mL of supplemented Schneider’ s medium. Slide smears from samples of the popliteal lymph node and the bone marrow, scrapes from the skin with lesions, and spleen and liver imprints were stained with Giemsa to search for amastigote forms of the parasite. *Leishmania* sp*.* culture and Giemsa staining were performed following the procedure described by Mendonça et al. (2017).

ELISA was performed using an EIE kit (Canine Visceral Leishmaniasis [Bio-Manguinhos, FIOCRUZ, Brazil]) with the following changes in the protocol described by the manufacturer: the blood serum was diluted at 1:400 and specific dog conjugate was replaced by A20-120P cat anti-IgG mouse conjugate at 1:20000. To determine cutoff points, we used serum samples from 10 control cats, obtained from the study by Mendonça et al. (2017), which were added to all plates. All 10 animals lived in the districts of Teresina, where canine VL had a low prevalence, and all were negative in the bone marrow PCR test. The cutoff point was calculated by averaging the optical density of negative controls plus three times the standard deviation value of OD from these negative controls.

### Hematological and biochemical quantification

All 60 animals included in the study underwent complete blood count and leukogram. For this purpose, 2 mL of blood was collected, both with 25 × 0.8 mm needles, into vacuum tubes with EDTA. Blood count was performed using an automatic counter (BC - 2800 Vet Mindray) with an ABX Vetpack kit and differential leukocyte count in a blood smear stained with panoptic fast stain.

The serum was obtained by collecting blood in a vacuum tube without anticoagulant and centrifuging at 1,600 rpm. It was then used to quantitate urea, creatinine, alanine aminotransferase (ALT), aspartate aminotransferase (AST), albumin, and total protein, using a semiautomatic device (Biochemical Analyzer BA 88 BIOCLIN) and Laboratory test kits as per the manufacturer’s recommendations. Globulin concentration was obtained by subtracting total protein from albumin.

### Histopathological diagnosis

For histopathological evaluation, five cats with VL, with their owner’s approval, underwent euthanasia under the following procedure: first, the cats were sedated with sodium thiopental 2.5% (80 mg/kg) and after confirming the absence of sensitivity and reflexes, they were humanely killed with 20% potassium chloride solution. Both were administered intravenously.

During the necropsy, a complete macroscopic evaluation was carried out. Spleen and liver aspirations, sown in NNN enriched with Schneider´s culture medium to search for *Leishmania* sp*.,* and collected fragments of kidneys, spleen, and liver, fixed in 10% buffered formalin solution for at least 24 hours, were used. These fragments then underwent a routine histological processing technique consisting of dehydration, diaphanization, imbibition, and inclusion of the tissue in paraffin, microtomy (4 μm), with subsequent dewaxing, hydration, hematoxylin and eosin staining, histological cut dehydration, and slide mounting. Histopathological changes were classified according to intensity (mild, moderate, and severe) (Lima et al., 2019) and distribution (absent, focal, multifocal, and diffuse) (Batista et al., 2020).

### Diagnosis of retroviruses

To assess the impact of immunosuppressive retroviruses, the animals with leishmaniasis were examined for feline leukemia virus (FeLV) antigen (p27) and IgG antibody against feline immunodeficiency virus (FIV). FeLV antigen and anti-FIV antibody detection were performed using a commercial assay kit (Alere FIV Ac/FeLV Ag Test Kit, Bionote Inc. 2-9 Seogu-dong, Hwaseong-si, Gyeonggi-do, Korea 440440), according to the manufacturer's recommendations.

### Statistical analysis

Hematological and biochemical parameters were analyzed using the Kruskal Wallis test and Dunns' post-test to verify the occurrence of significant differences between the three groups (infected, suspect, and control). The Mann-Whitney test was also performed to analyze possible significant differences in the number of clinical signs between FIV positive and negative animals. Statistical tests were performed using GraphPad Prism 5.0 program (GraphPad Software Inc., San Diego, CA, USA) and assuming a 5% error probability.

## Results

**Figure 1:**
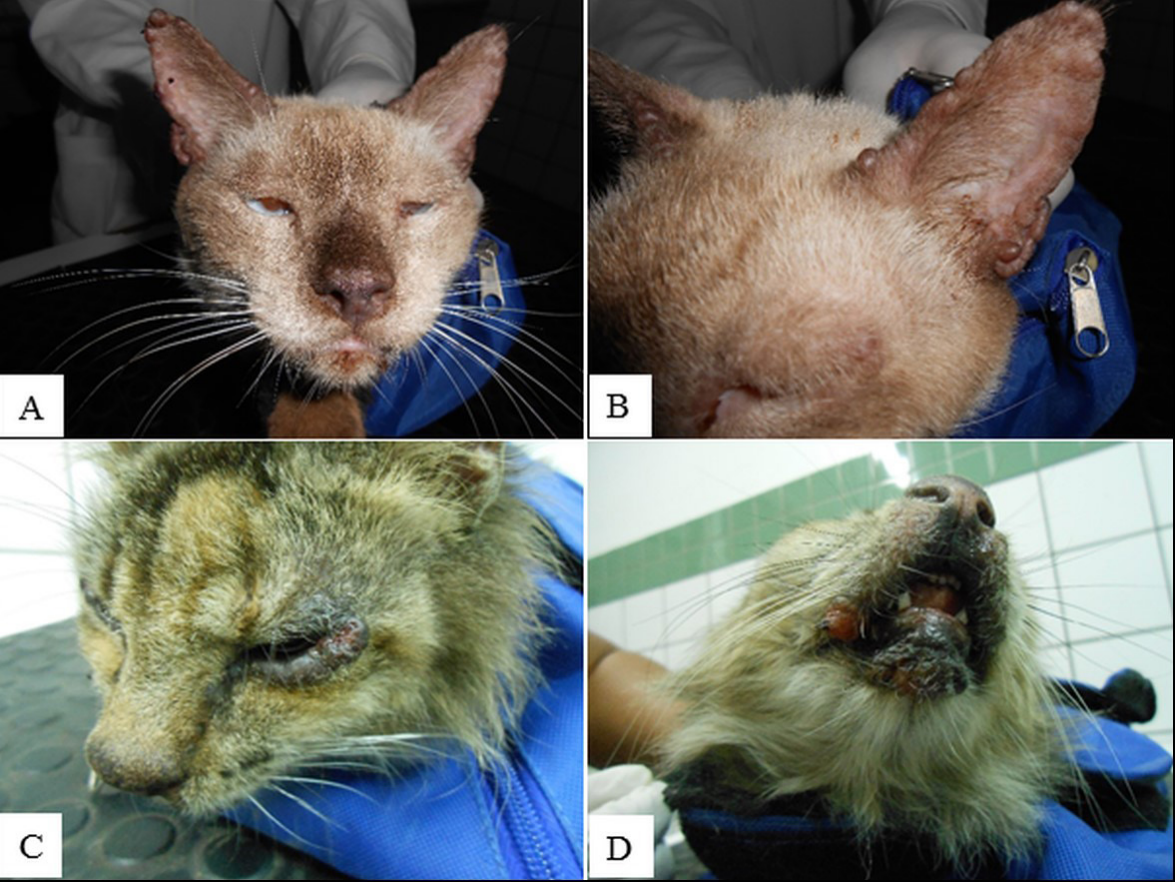
Clinical changes observed in cats infected with *L. infantum*. A and B - Nodules with a soft consistency at the edges of both ears (bilateral). C - Blepharitis and periocular lesion. D - Nodule that evolved to ulcerative lesions at the upper lip.

Clinical evaluation revealed that, out of 20 cats infected by *L. infantum*, only two (10%) did not present any clinical alteration. The most frequent changes in animals with leishmaniasis were lymphadenomegaly (65%), alopecia (55%), ulcerative skin lesions and weight loss (40%), and skin nodules (25%) (Table 1). A total of 14 animals (70%) had more than one clinical alteration and skin lesions were more frequently located in the head region, especially in the ears, periocular region, muzzle, and lips (Table 1) (Figure 1).

**Table 1:** Number and percentage of clinical signs in cats from the infected group (animals with leishmaniasis) and suspected group (animals with clinical alterations or positive to serology and negative to parasitological exam for leishmaniasis).

Characteristics	Infected n=20 (%)	Suspected n=20 (%)
Lymphadenomegaly	13 (65)	14 (70)
Weight loss	8 (40)	4 (20)
Skin lesions	13 (65)	10 (50)
Alopecia	11 (55)	8 (40)
Ulcerative lesions	8 (40)	4 (20)
Nodules	5 (25)	0 (0)
Ocular lesions	4 (20)	1 (5)
Uveitis	3 (15)	0 (0)
Blepharitis	2 (10)	0 (0)
Eye discharge	1 (5)	1 (5)
Blindness	1 (5)	0 (0)
Dehydration	1 (5)	1 (5)
Asymptomatic	2 (10)	1 (5)

**Figure 2:**
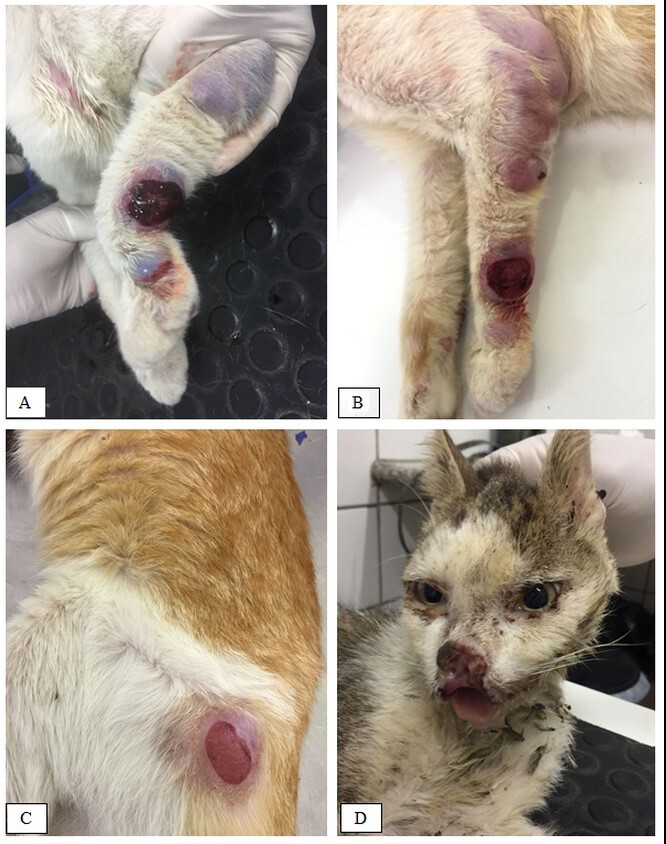
Nodules and lesions in the skin of infected cats with *L. infantum*. A and B - Nodules and lesions in legs. C - Skin lesion close to the acetabulofemural region. D - Lesion involving muzzle, nasal plane, and upper lip

**Figure 3:**
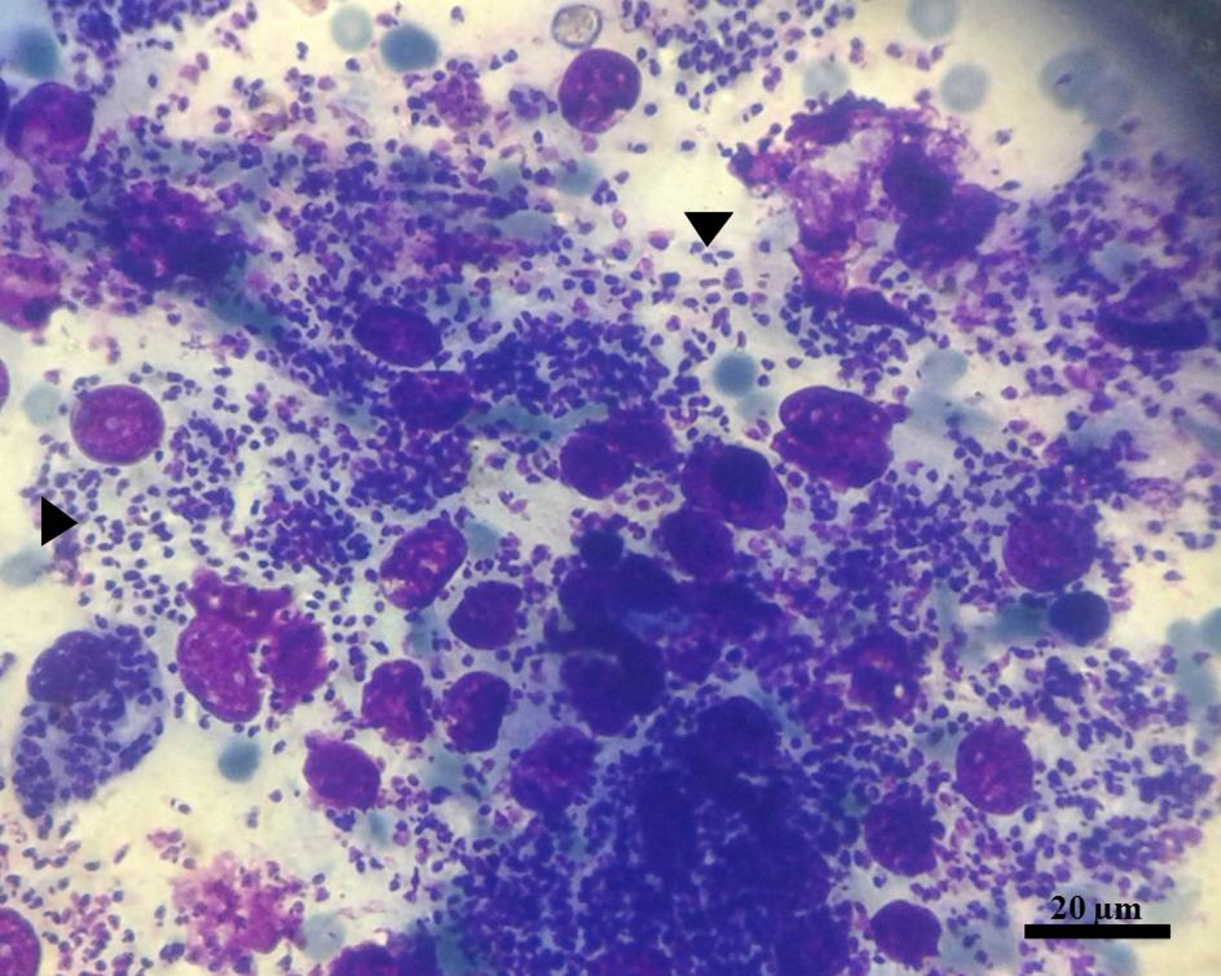
Amastigote forms of *Leishmania* sp*.* (arrows) in smear stained with Giemsa, performed using bloody exudate sample of a nodule located at the pinnae of a cat infected by *L. infantum.*

As for skin lesions, nodules of varied dimensions were observed, ranging from a few mm to approximately 5 cm in diameter (Figure 1A, 1B, 2A, and 2B). As the disease progressed, nodules ulcerated (Figure 2), and when smearing the slides with the nodule exudate, it was possible to observe a large amount of amastigote forms of *Leishmania* sp*.* (Figure 3).

A total of 20 animals with *L. infantum* were also tested for retrovirosis. None were positive for FeLV, while seven (35%) were positive for FIV. When assessing the interference of FIV infection in the occurrence of clinical signs in animals infected with *L. infantum*, there was no significant difference in the number of clinical signs in positive and negative animals for the Mann-Whitney test (p = 0,2300).

When the results of blood count and biochemical tests were analyzed, animals with VL had significantly lower red blood cell (p = 0.0005) and hematocrit (p = 0.0007) concentrations when compared to values obtained from animals belonging to the control and suspected groups, and creatinine concentrations of the infected group were lower than those of the control group (p = 0.0073) (Kruskal Wallis test, Dunns post-test) (Table 2).

**Table 2:** Mean and standard deviation of hematological and biochemical parameters in cats without clinical signs and negative for visceral leishmaniasis (VL) tests (control group), with clinical alteration or positive serology for VL and negative by direct smear for *Leishmania* sp. (suspected group) and for those with VL (infected group).

Parameters	Control n=20	Suspects n=20	Infected n=20	Reference values
Red blood cell (x 10^6^/μL)	7.0 ± 0.8^a^	7.1 ± 1.4^a^	5.6 ± 1.4^b^	5 - 10*
Hemoglobin (g/dL)	10.3 ± 1.2^a^	10.8 ± 2.1^a^	9.4 ± 1.9^a^	8 - 15*
Hematocrit (%)	31.5 ± 3.3^a^	32.4 ± 5.9^a^	26.6 ± 5.4^b^	24 - 45*
MCV (fL)	45.5 ± 2.9^a^	46.0 ± 2.7^ab^	48.9 ± 4.5^b^	39 - 55*
MCHC (%)	32.6 ± 3.5^a^	33.3 ± 2.1^a^	35.8 ± 6.8^a^	30 - 36*
Platelets (x10^3^/*µL*)	282.3 ± 114.9^a^	386.3 ± 141.2^a^	300.1 ± 198.5^a^	200 - 800*
Total leukocytes	15500 ± 8101^a^	18610 ± 8052^a^	15980 ± 6696^a^	5500 - 19000*
Segmented	8758 ± 6187^a^	13080 ± 8173^a^	10390 ± 5494^a^	2500 - 12500*
Lymphocytes	5297 ± 3572^a^	2402 ± 1698^b^	4212 ± 4037^ab^	1500 - 7000^*^
Urea (mg/dL)	54.6 ± 14.0^a^	50.1 ± 10.7^a^	50.3 ± 28.5^a^	20 - 30^**^
Creatinine (mg/dL)	1.4 ± 0.4^a^	1.2 ± 0.4^ab^	1.0 ± 0.4^b^	0.8 - 1.8**
Total Protein (g/dL)	8.3 ± 2.2^a^	8.6 ± 2.0^a^	8.9 ± 1.6^a^	5.4 - 7.8**
Albumin (g/dL)	2.1 ± 0.5^a^	2.0 ± 0.5^a^	2.0 ± 0.5^a^	2.1 - 3.3**
Globulin (g/dL)	6.0 ± 1.9^a^	6.6 ± 2.2^a^	6.8 ± 1.9^a^	2.6 - 5.1**
A/G Ratio (g/dL)	0.4 ± 0.1^a^	0.4 ± 0.2^a^	0.3 ± 0.2^a^	0.45 - 1.19**
ALT (U/L)	59.2 ± 46.1^a^	52.9 ± 31.0^a^	50.7 ± 49.8^a^	8 - 88**
AST (U/L)	33.3 ± 13.1^a^	41.1 ± 28.1^a^	53.0 ± 92.2^a^	26 - 43**

* (Jain, 1993);

** (Kaneko et al., 1997).

Legend: MCV, mean corpuscular volume; MCHC, mean corpuscular hemoglobin concentration; ALT, alanine aminotransferase; AST, aspartate aminotransferase. Compact letter display indicates significant difference between distinct groups in the same row (Kruskal-Wallis test, Dunns post-test, p <0.05).

Six (30%) of the 20 animals with VL had erythrocyte values below the reference values as described by Jain (1993). In five (25%) animals, anemia was the normocytic normochromic type and in one (5%) anemia was hypochromic.

In addition to clinical, hematological, and biochemical evaluations, a necropsy was performed on five animals infected with *L. infantum*. The most frequently observed macroscopic changes were widespread lymphadenomegaly (multiple lymph nodes visually larger than those normally seen in cats), which was present in all five necropsied animals. Regarding microscopic lesions, it was observed that hyperemia was often present in the spleen (100%), liver (60%), and kidney (80%). In the spleen and liver, the presence of *Leishmania* sp*.* was also observed, certifying the parasite visceralization in four of five animals that underwent necropsy (Table 3).

**Table 3:** *Leishmania* sp*.* occurrence in liver and macroscopic changes observed during necrops y and microscopic changes observed in histopathology of liver and kidney in cats infected with *L. infantum.*

Assessed Parameters	Animal					Total (%)
Leishmania sp. search	I	II	III	IV	V	
Spleen	–	+	+	–	+	
Liver	–	–	–	+	+	
Macroscopic changes						
Widespread lymphadenomegaly	+	+	+	+	+	5 (100)
Pale mucosae	+	+				2 (40)
Splenomegaly	+				+	2 (40)
White pulp hyperplasia					+	1 (20)
Petechiae diffusely distributed in lung	+					1 (20)
Cyanosis	+					1 (20)
Discrete multifocal pneumonia					+	1 (20)
Microscopic changes						
Spleen						
Hyperemia	+	+	+	+	+	5 (100)
White pulp hyperplasia	+	+		+	+	4 (80)
Red pulp hyperplasia		+				1 (20)
Leishmania sp. in macrophages		+			+	2 (40)
Liver						
Hyperemia	+			+	+	3 (60)
Lymphoplasmacytic hepatitis	+			+		2 (40)
Lymphocytic hepatitis		+				1 (20)
Liver degeneration	+		+	+	+	4 (80)
Kidneys						
Hyperemia	+		+	+	+	4 (80)
Intersticial nefritis			+			1 (20)
Proliferative Glomerulonephritis	+				+	2 (40)
Membranoproliferative glomerulonephritis			+			1 (20)

Subtitle: + indicates the occurrence of pathological changes in the animal or the presence of *Leishmania* sp*.* and - indicates a negative result in *Leishmania* sp*.* search.

Regarding the histopathological changes, mild hepatitis was observed, with the distribution ranging from focal to diffuse and liver degeneration varying from mild to moderate and multifocal to diffuse. In kidneys, interstitial nephritis and proliferative glomerulonephritis were mild and multifocal, and membranoproliferative glomerulonephritis was moderate and multifocal.

## Discussion

A previous study reported the occurrence of VL in cats in Teresina, Piauí, Brazil (Mendonça et al., 2017). However, little is known about the clinical changes occurring in cats affected by the disease. This is mainly due to the small number of positive animals found in the study, limiting the information and thus, underreporting the lesions occurring in VL in cats. As for the clinical manifestations observed in the animals included in this study, changes in the skin were present at a high frequency. Alopecia, ulcerative skin lesions, and skin nodules appeared with a frequency of 52.6%, 36.8%, and 26.3%, respectively. Other studies in different regions of Brazil have also reported a high frequency of skin lesions in cats with VL (Navarro et al., 2010; Vides et al., 2011). These results suggest a possible high parasitic load on the skin and a possible source of infection for *L. longipalpis* sand flies. Studies have already reported of cats with *L. infantum* having a high capacity to infect *L. longipalpis* (Silva et al., 2010; Mendonça et al., 2020).

A curious observation in two of the 20 infected animals was that the nodules appearing at the skin edges of the ears were flaccid, containing bloody exudate and macrophages full of amastigote forms of *Leishmania* sp. In three other animals, although the nodules were hardened, they were also hemorrhagic and with an enormous amount of *Leishmania* sp. amastigotes. As the disease progressed, the nodules ulcerated and while some pet caregivers implemented a topical treatment of the lesions using antimicrobial ointment, the results were unsatisfactory.

Studies related to VL in cats have reported their natural resistance to infection by *L. infantum* (Kirkpatrick et al., 1984; Costa et al., 2010). Besides, *Leishmania* co-infection with retroviruses FIV and FeLV is common, as they are viruses interfering with the immune response of cats, making them more susceptible to other infections (Simões-Mattos et al., 2005; Costa et al., 2010; Abramo et al., 2021). In this study, the analysis to verify *Retrovirus* infection interference in the number of clinical signs showed that FIV infection did not significantly favor an increase in the number of clinical signs, and no animal was positive for FeLV.

This is the first study to make a broad assessment of possible hematological and biochemical changes, comparing a group of infected animals with a group composed of clinically healthy animals, and it is possible to observe that anemia is frequent in cats with VL. In addition, nine other animals were not considered anemic according to reference values described by Jain (1993); however, these animals presented values below the average observed in the healthy animals’ group. Several other studies on the occurrence of anemia in dogs with VL reported findings similar to ours, i.e., the most common type of anemia was normocytic normochromic (Aguiar et al., 2007; Ikeda-Garcia et al., 2008; Medeiros et al., 2008; Mendonça et al., 2015).

A study by Nicolato et al. (2013) in dogs with severe VL reported the association of anemia with a disorder in the erythroid bone marrow compartment, with reduced red blood cell count, a mechanism that needs further studies to confirm the occurrence in feline leishmaniasis. Another possible cause of anemia is increased hemolysis in blood and liver, which is associated with an inflammatory response to infection by *L. infantum* (Saeed et al., 1998). Furthermore, decreased plasma iron in the presence of greatly increased iron storage suggested that the association of reticuloendothelial hyperplasia with abnormal iron retention by macrophages, typical of anemia in chronic disorders, limiting the erythropoietic response to anemia in chronic VL (Pippard et al., 1986).

As for the biochemical quantifications, the results do not point out significant differences by comparing infected to non-infected animals, indicating that the possible presence of lesions in visceral organs, in general, was not severe enough to change the biochemical parameters evaluated in this study. The results of histopathological analysis detected the presence of lesions in the liver, spleen, and kidney and the occurrence of the parasite in the spleen and liver, confirming the visceralization of *L. infantum* in cats. The non-detection of parasites in the kidneys suggests the development of VL renal lesions in cats similar to that occurring in dogs, caused not by the presence of parasites, but by exposure to immune complexes. This induces the formation of inflammatory infiltrates, such as proliferative and membranoproliferative glomerulonephritis, observed in the cats in this study and observed in dogs with VL (Nieto et al., 1992; Alves et al., 2013).

In the findings of this study, it was observed that inflammatory infiltrates in visceral organs are frequently present in cats infected with *L. infantum*, as also reported in other studies on canine and feline leishmaniasis (Navarro et al., 2010; Andrade et al., 2014; Batista et al., 2020). However, other studies will be necessary to investigate the pathogenesis of visceral alterations in cats with leishmaniasis, as well as to investigate the possible relationship between the occurrence of hyperemia in the spleen, liver, and kidneys of cats with leishmaniasis, since hyperemia is not a microscopic alteration commonly found in other cases of canine and feline leishmaniasis (Navarro et al., 2010; Batista et al., 2020).

## Conclusions

Clinical changes are very common in cats with leishmaniasis, especially lymphadenomegaly, weight loss, and skin lesions located mainly in the head region. Observation of these clinical manifestations, along with the changes in blood count parameters, contributes significantly to the diagnosis of the disease in cats and is perfectly feasible for clarifying disease progression.

As for biochemical parameters, there is a statistical similarity between animals with VL and clinically healthy and negative animals in serological and parasitological examinations for leishmaniasis. It can be concluded that serum biochemistry assessment, as a single test, does not provide enough information for clinicians to suspect *L. infantum* infection in cats.

In the evaluation of organs such as the spleen, liver, and kidneys, the visceralization of *L. infantum* in cats was confirmed, and histopathological analysis of these organs revealed a high frequency of lesions suggestive of VL.
